# Sequence analysis of annually normalized citation counts: an empirical analysis based on the characteristic scores and scales (CSS) method

**DOI:** 10.1007/s11192-017-2521-9

**Published:** 2017-09-19

**Authors:** Lutz Bornmann, Adam Y. Ye, Fred Y. Ye

**Affiliations:** 1 Division for Science and Innovation Studies, Administrative Headquarters of the Max Planck Society, Hofgartenstr. 8, 80539 Munich, Germany; 2 Center for Bioinformatics, School of Life Sciences, Peking University, Beijing, 100871 China; 3 Jiangsu Key Laboratory of Data Engineering and Knowledge Service, Nanjing University, Nanjing, 210023 China

**Keywords:** Citation analysis, Sequence analysis, Annually normalized citations, Dynamically normalized impact counts (DNIC), Characteristic scores and scales (CSS)

## Abstract

In bibliometrics, only a few publications have focused on the citation histories of publications, where the citations for each citing year are assessed. In this study, therefore, annual categories of field- and time-normalized citation scores (based on the characteristic scores and scales method: 0 = poorly cited, 1 = fairly cited, 2 = remarkably cited, and 3 = outstandingly cited) are used to study the citation histories of papers. As our dataset, we used all articles published in 2000 and their annual citation scores until 2015. We generated annual sequences of citation scores (e.g., $\left\{ {01233233221} \right\}$) and compared the sequences of annual citation scores of six broader fields (natural sciences, engineering and technology, medical and health sciences, agricultural sciences, social sciences, and humanities). In agreement with previous studies, our results demonstrate that sequences with poorly cited (0) and fairly cited (1) elements dominate the publication set; sequences with remarkably cited (3) and outstandingly cited (4) periods are rare. The highest percentages of constantly poorly cited papers can be found in the social sciences; the lowest percentages are in the agricultural sciences and humanities. The largest group of papers with remarkably cited (3) and/or outstandingly cited (4) periods shows an increasing impact over the citing years with the following orders of sequences: $\left\{ {0123} \right\}$ (6.01%), which is followed by $\left\{ {123} \right\}$ (1.62%). Only 0.11% of the papers (*n* = 909) are constantly on the outstandingly cited level.

## Introduction

Bibliometrics is the backbone of scientometrics; most of the studies in scientometrics are based on publication and citation data (Vinkler 2016). Bibliometrics applies statistical methods for analyzing counts of publications and citations (University of Waterloo Working Group on Bibliometrics 2016). Since the introduction of citation analysis (Garfield 1955), citations have been seen as the basic unit of impact which follow from “votes” of citing authors for publications (Bornmann and Marx 2014; Jha et al. 2016). “The act of citing another person’s research provides the necessary linkages between people, ideas, journals and institutions to constitute an empirical field or network that can be analysed quantitatively” (Mingers and Leydesdorff 2015, p. 1). Many publications in bibliometrics have focused on analyzing the distributions of citations. For example, Albarrán and Ruiz-Castillo (2011) investigated 3.7 million articles published in 22 scientific fields. They found that “citation distributions are highly skewed: About 70% of all articles receive citations below the mean, and articles with a remarkable or outstanding number of citations represent about 9% of the total” (p. 48). According to the results of Ponomarev et al. (2012), “a typical citation pattern has an initial period of slow citation growth lasting from 5 to 20 months… After this initial slow growth phase, the citation rates accelerate until they reach saturation plateaus, after which they decrease”.

However, there is a gap in the literature with respect to studies analyzing citation distributions in more detail. In this study, therefore, annual categories of normalized citation scores (“poorly cited”, “fairly cited”, “remarkably cited”, and “outstandingly cited”) are used to study the citation histories of papers (Glänzel and Schubert 1988). As our dataset, we use all the articles published in 2000 and their annual citation scores until 2015. We compare the sequences of annual citation scores in six broader fields (natural sciences, engineering and technology, medical and health sciences, agricultural sciences, social sciences, and humanities).

## Literature overview

An early study with the focus on number of citations as a function of time was published by Vlachy (1985). The aging of information in papers (measured by synchronous or diachronous methods) have been studied by Glänzel and Schubert (1995) as well as Glänzel (1997, 2004). Schubert and Glänzel (1986) introduced the so called “response time” which reveals the speed of receiving citation impact (see also Bornmann and Daniel 2010). They found different times between the fields.

Only a few studies have focused on the citation histories of publications, where the citations for every year are assessed (whether they are lower or higher compared to citations which other publications received in the same year). Most of these studies have dealt with specific distributions of citations. Good examples are sleeping beauties. These are papers which generate little or no citation impact over a long time period (e.g. 10 years), before they start to generate considerable impact. According to Mir and Ausloos (2016), the phenomenon of sleeping beauties is also labeled as resisted discoveries, premature discoveries, delayed recognition, or information awakening. Overviews on sleeping beauties’ studies can be found in Teixeira et al. (2016) and Min et al. (2016).

Recently, the citation histories of papers have been investigated in more detail by two studies. Baumgartner and Leydesdorff (2014) explored the citation curves (1) of six journals in different fields as well as (2) in one entire field (virology) over 16 years. Basically, they found two typical curves: “sticky knowledge claims” continue to be cited more than 10 years after publication. “Transient knowledge claims” show a decay pattern after reaching an early peak. The other study by Colavizza and Franceschet (2016) investigated the *Physical Review* archive, covering 120 years of physics. They found the following three types of citation curve: “(1) Marathoners: publications which start fast or slow, reach a moderate peak and keep improving the ratio of received citations, or at least keep being relevant over prolonged amounts of time by manifesting a slow decline or a plateau. Marathoners in effect tend to age slowly, or not at all, and are also more numerous and varied than sprinters. (2) Sprinters: publications with fast, even extremely fast and high peak, and equally rapid ageing. These publications are immediately relevant for their community, and rapidly forgotten thereafter, and are fewer in number in the APS dataset. (3) Middle-of-the-roads: publications with a citation history close to the global average citation history, that is, a fast but moderately peaking curve with a gradual decay over time” (p. 1043).

## Methods

### Field normalization of citation impact

This study uses standard impact scores in bibliometrics, namely field- and time-normalized citation impact scores (in a dynamical variant) (Vinkler 2010). These dynamically normalized impact counts (DNIC) are defined as

1 $${\text{DNIC}}_{ij} = \frac{{C_{ij} }}{{E_{fj} }},\quad f = f(i)$$

2 $$E_{fj} = \frac{1}{{N_{fj} }}\sum\limits_{{i\left| {f = f(i)} \right.}} {C_{ij} }$$

where *i* = 1, 2,… are publications, *j* = 1, 2,… are citing years, and *f* = 1, 2,… are fields. Here, field delineations based on disciplinary OECD minor codes are used. The OECD field definitions can be found at http://www.oecd.org/science/inno/38235147.pdf. We selected the 2 digit level scheme.

*C*_*ij*_ denotes citations received by publication *i* in year *j,* and *E*_*fj*_ denotes mean (received) citations of all publications in field *f* and year *j* (i.e. *E*_*fj*_ is the expected value). *N*_*fj*_ is the number of cited publications in field *f* and year *j* (*N*_*fj*_ is based on non-zero citations), and *f* = *f*(*i*) means a certain field of a given publication. The indicator follows the standard approach in bibliometrics with both field- and time-normalized citations (Waltman 2016). The difference from the standard approach in bibliometrics is that the calculation is based on annual citations, and not on the citations between publication year and a fixed time point later on.

If *C*_*ij*_ = 0, then DNIC_*ij*_ = 0. If DNIC_*ij*_ > 1, the citation impact of the publication is higher than the average in the corresponding OECD disciplinary category and (cited as well as citing) publication years. If DNIC_*ij*_ < 1, the impact is lower than the average.

### Classifying of publications using the CSS method

Glänzel and Schubert (1988) introduced the characteristic scores and scales (CSS) method for grouping ranked observations into rank-specific categories (see also Glänzel 2007, 2010, 2011). Consider a set of *n* papers. The observed citations *X*_*i*_ received by paper *i* are ranked in descending order, $X_{1}^{*} \ge X_{2}^{*} \ge \ldots \ge X_{n}^{*}$, where *X*_1_^*^ and *X*_n_^*^ denote the citations of the most and least frequently cited papers, respectively. Set the initial values *β*_0_ = 0 and *v*_0_ = *n*, where *n* is the number of papers. *β*_*1*_ is defined as the mean citations; *v*_*1*_ is defined by the comparison $X_{{v_{1} }}^{ * } \ge \beta_{1}$ and $X_{{v_{1} + 1}}^{ * } < \beta_{1}$. This comparison is repeated, yielding

3 $$\beta_{k} = \sum\limits_{i = 1}^{{v_{k - 1} }} {\frac{{X_{i}^{*} }}{{v_{k - 1} }}} \quad {\text{with}}\quad X_{{v_{k} }}^{*} \ge \beta_{k} \quad {\text{and}}\quad X_{{v_{k} + 1}}^{*} < \beta_{k} ,\quad {\text{for }}k \ge 2$$

Thus, we obtain series *β*_*0*_ ≤ *β*_*1*_ ≤ … and *v*_*0*_ ≥ *v*_*1*_ ≥ …. The *k*th class is defined by the pair of threshold values [*β*_*k*−1_, *β*_*k*_]; the number of papers belonging to this class amounts to *v*_*k*−1_ − *v*_*k*_.

The CSS method can be used to classify the papers within certain fields into four impact classes: “poorly cited”, “fairly cited”, “remarkably cited”, and “outstandingly cited”. Then, for example, the share of outstandingly cited papers can be determined for a set which includes papers from different fields (e.g. all papers published by a university). However, the method can not only be used to classify single papers, but also to certain aggregates of papers. For example, Bornmann and Glänzel (2017) propose using the CSS method to classify the universities in a specific ranking (e.g. the Leiden ranking) into performance classes (e.g. based on the number of highly-cited papers). The universities can then be separated into low and high performers.

In this study, we use the CSS method for classifying the papers into four citation impact classes based on DNIC_*ij*_. Thus, we do not use the citation counts of single papers, but the annual field- and time-normalized scores for the classification. Consider the set $\left\{ {{\text{DNIC}}_{ij} } \right\}$ of *n* papers published in various disciplines. We used the OECD major codes to compare the results of six broad disciplines: natural sciences, engineering and technology, medical and health sciences, agricultural sciences, social sciences, and the humanities. The broad disciplines are aggregates of OECD minor codes.

In each discipline and across disciplines, the DNIC_*ij*_ scores (of paper *i* in a given year *j*) are ranked in descending order (${\text{DNIC}}_{1}^{ * } \ge {\text{DNIC}}_{2}^{ * } \ge \ldots \ge {\text{DNIC}}_{n}^{ * }$)_*j*_. The comparison between DNIC and *β* is defined by

4 $$\beta_{kj} = \sum\limits_{i = 1}^{{v_{k - 1} }} {\frac{{{\text{DNIC}}_{ij}^{ * } }}{{v_{k - 1} }}} ,\quad {\text{DNIC}}_{{v_{k} j}}^{ * } \ge \beta_{kj} \quad {\text{and}}\quad {\text{DNIC}}_{{v_{k} j + 1}}^{ * } < \beta_{kj}$$

Then, the pair of threshold values [*β*_*k*−1_, *β*_*k*_] forms the impact class. Using the CSS method, the annual categorization of papers to citation impact classes is based therefore on the annual DNIC scores. The values of the annual DNIC scores are kept with min *k* ≥ 2, 3, …, respectively, which means *k* ≥ 2, 3, … in every year after the publication year. Since the values *k* = 2 and *k* = 3 are usually used to identify highly cited papers (Glänzel 2011), we set *k* ≥ 2 as “fairly cited” papers, *k* ≥ 3 as “remarkably cited” papers, and *k* ≥ 4 as “outstandingly cited” papers in the long run.

### Sequence analysis of annual CSS scores

**Fig. 1:**
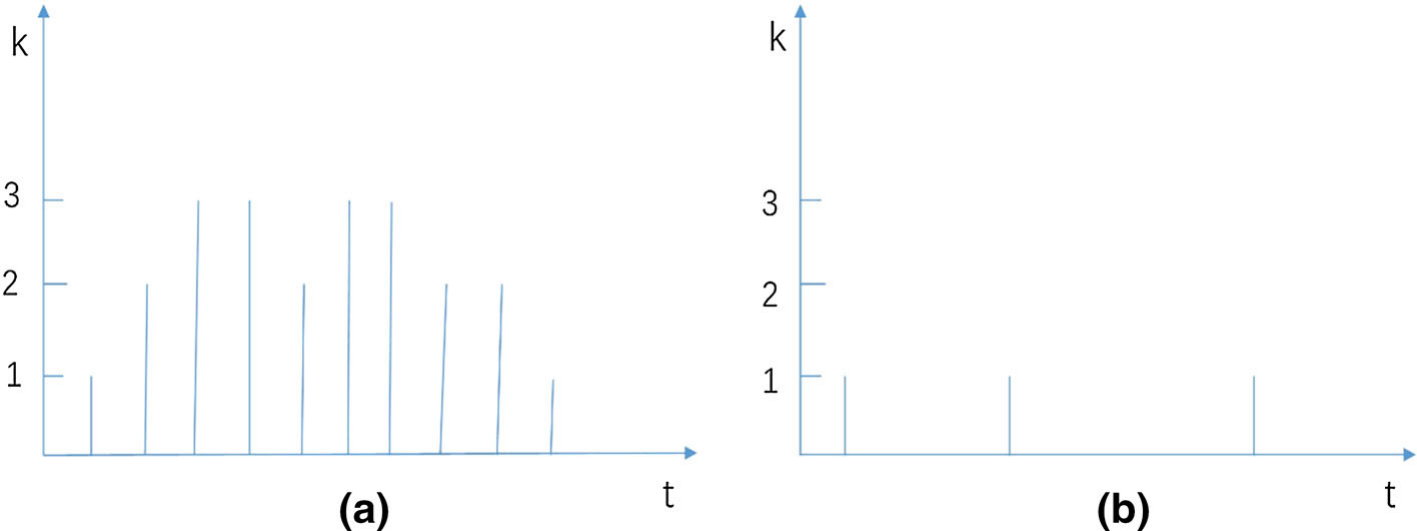
Two examples of CSS score sequences

In a yearly time series *j* = 1, 2,…, *m*, the annual CSS scores *k* of each publication form a sequence across 16 years (starting in 2000). In other words, we have a sequence of 16 scores for every publication with values between 0 = poorly cited and 4 = outstandingly cited. Two examples of sequences are shown in Fig. 1. Sequence $\left\{ a \right\}$ is $\left\{ {01233233221} \right\}$ and sequence $\left\{ b \right\}$ is $\left\{ {01001000100} \right\}$. $\left\{ a \right\}$ indicates a highly cited publication (most of the time) and $\left\{ b \right\}$ a constantly little cited or non-cited publication.

The statistical analyses of the data in the current study are based on the strategy proposed by Brzinsky-Fay et al. (2006) for the analysis of sequence data. Sequence data is analyzed in many research fields, e.g. DNA sequences in biology and life courses in social sciences. “A sequence is defined as an ordered list of elements, where an element can be a certain status (e.g., employment or marital status), a physical object (e.g., base pair of DNA, protein, or enzyme), or an event (e.g., a dance step or bird call). The positions of the elements are fixed and ordered by elapsed time or by another more or less natural order” (Brzinsky-Fay et al. 2006, p. 435).

### Dataset used

The bibliometric data used in this study is from an in-house database developed and maintained by the Max Planck Digital Library (MPDL, Munich) and derived from the Science Citation Index Expanded (SCI-E), Social Sciences Citation Index (SSCI), and Arts and Humanities Citation Index (AHCI) prepared by Clarivate Analytics, formerly the IP & Science business of Thomson Reuters. The study is based on 790,698 articles published in 2000 and the corresponding citations across 16 citing years (with 2000 as the first citing year). Since many papers have been assigned to more than one OECD minor code, 161,302 papers appear between two and six times in the dataset (435,634 papers have no duplicates). We decided to let the papers appear multiple times in the dataset, since the papers might have different citation distributions in the disciplines.

Table 1 shows the number of annual CSS categories in the dataset. Since we included 790,698 articles with 16 annual citation scores each in the study, the study is based on 12,651,168 annual CSS categories.

**Table 1:** Number of annual CSS categories in the dataset (16 categories per article)

CSS categories	Absolute number	In percent	Cumulative relative number
Poorly cited (0)	8,956,874	70.80	70.80
Fairly cited (1)	2,642,053	20.88	91.68
Remarkably cited (2)	753,340	5.95	97.64
Outstandingly cited (3)	298,901	2.36	100.00
Total	12,651,168	100.00	

## Results

### Descriptive statistics

The sequence analyses which we describe in the “Sequence analysis” section are based on several transformations of the original raw data from the MPDL in-house database. In order to reveal the relations between the raw data and the transformed (field- and time-normalized) data, Table 2 shows annual citations, annual normalised citation scores (DNIC), and sequences of CSS scores for some example papers.

**Table 2:** Examples for demonstrating the relationship between annual citations, annul normalized citation scores (DNIC), and sequences of CSS scores

Group	Web of science accession number	OECD	Annual citations	Annual normalized citation scores (DNIC)	Sequence of CSS scores
(1)	000086005500004	5.09	0,0,0,0,0,1,1,1,1,4,2,5,9,7,7,6	0,0,0,0,0,0.45,0.43,0.40,0.36,1.43,0.65,1.5,2.6,2.1,2.1,1.7	0000000001122222
	000087093700005	5.07	0,0,0,0,0,2,2,4,3,3,2,5,6,9,10,7	0,0,0,0,0,0.88,0.76,1.5,1.06,1.01,0.64,1.6,1.9,2.6,2.9,2.0	0000011111122222
	000086306700004	5.01	0,0,0,2,1,4,6,6,8,9,13,8,9,7,11,9	0,0,0,0.64,0.30,1.2,1.6,1.6,2,2.2,3.1,1.9,2.1,1.5,2.4,2.0	0000011122222222
	000165574800007	5.02	0,0,0,0,0,0,3,7,12,18,16,25,19,29,29,22	0,0,0,0,0,0,0.91,1.9,2.9,4.1,3.6,5.5,4.0,5.9,5.8,4.4	0000001223333333
	000088345800017	2.08	0,0,0,0,0,0,1,0,1,3,5,12,15,13,21,13	0,0,0,0,0,0,0.27,0,0.27,0.84,1.4,3.3,4.2,3.7,6.1,3.9	0000000001133333
	000166239900005	2.08	0,0,0,0,0,0,0,0,1,3,5,13,13,22,17,14	0,0,0,0,0,0,0,0,0.27,0.84,1.4,3.6,3.7,6.2,5,4.2	0000000001133333
	000165810100004	2.05	0,1,1,1,1,1,2,7,9,7,11,14,13,15,15,15	0,0.44,0.36,0.34,0.33,0.32,0.62,2.2,2.8,2.2,3.4,4.3,4,4.6,4.6,4.6	0000001222333333
	000165369700102	1.03	0,1,1,2,2,0,0,1,6,9,8,13,14,19,17,13	0,0.28,0.26,0.52,0.53,0,0,0.28,1.7,2.5,2.3,3.7,4,5.5,4.9,3.9	0000000012233333
	000089390700042	1.07	1,13,11,11,19,25,35,36,42,43,45,44,65,45,50,49	0.22,1.1,0.74,0.75,1.3,1.8,2.5,2.6,3.1,3.3,3.5,3.5,5.2,3.6,4.2,4.4	1111122222333333
	000088767700039	1.07	3,14,19,23,24,24,28,52,47,49,51,76,44,58,77,66	0.67,1.2,1.3,1.6,1.6,1.7,2,3.8,3.5,3.8,4,6,3.5,4.7,6.5,5.9	1111122333333333
(2)	000086625000041	1.07	6,18,28,36,26,36,39,39,34,27,30,24,38,33,35,26	1.3,1.5,1.9,2.5,1.8,2.5,2.8,2.8,2.5,2.1,2.4,1.9,3,2.7,3,2.3	2222222222222222
	000085941400041	1.07	8,22,35,41,44,38,30,31,33,32,25,31,34,29,31,27	1.8,1.8,2.4,2.8,3,2.7,2.1,2.3,2.4,2.5,2,2.5,2.7,2.3,2.6,2.4	2222222222222222
	000087782900061	1.07	5,32,34,39,32,38,36,38,43,27,29,30,35,27,35,30	1.1,2.6,2.3,2.7,2.2,2.7,2.6,2.8,3.2,2.1,2.3,2.4,2.8,2.2,3,2.7	2222222222222222
	000086951000033	3.02	5,11,15,16,14,10,16,19,23,19,12,15,14,16,14,17	2.7,3.3,3.7,3.9,3.5,2.5,4.1,4.9,6,5.1,3.3,4.2,3.9,4.5,4.1,5.1	3333323333333333
(3)	000084896300080	1.06	6,14,16,14,22,32,11,7,12,4,1,0,1,1,0,0	2.8,3.6,3.5,3.1,5,7.5,2.7,1.7,3,1,0.27,0,0.27,0.28,0,0	3333342221000000
(4)	000085121500021	1.06	1,8,7,5,11,4,7,4,11,6,12,4,7,4,7,8	0.47,2,1.6,1.1,2.5,0.94,1.7,0.99,2.8,1.6,3.2,1.1,1.9,1.1,2,2.3	1211212121212122
	000089349800014	1.05	0,8,10,8,10,11,5,13,4,12,5,9,12,6,13,7	0,3.5,3.5,2.5,3.1,3.4,1.5,3.8,1.2,3.4,1.4,2.6,3.3,1.6,3.5,1.9	0332231313123232

Four groups of examples are presented: papers with (1) increasing, (2) stable, (3) decreasing, and (4) fluctuating citation impact across the years

Table 2 tries to demonstrate the spectrum of different citation impact histories in the dataset. Group (1) in the table consists of papers with increasing citation impact over the citing years. The citation impact of the papers in group (2) is more or less stable over the years. Decreasing and fluctuating histories, respectively, are shown under group (3) and (4) in the table. The WoS accession numbers listed can be used to inspect the paper and its citations in WoS in more detail.

The CSS method was initially proposed by Glänzel and Schubert (1988). Since then, the method has been used in various contexts to classify single papers or aggregates of papers as “poorly cited”, “fairly cited”, “remarkably cited”, and “outstandingly cited” (Albarrán and Ruiz-Castillo 2011; Bornmann and Glänzel 2017; Glänzel 2007, 2010, 2011; Li et al. 2013). Although the studies were based on different bibliometric datasets, the distributions seem to follow (more or less) a general distribution pattern of percentages: 70% (poorly cited)—21% (fairly cited)—7% (remarkably cited)—2% (outstandingly cited). In addition, similar distribution patterns are reported by Chi and Glänzel (2016) in the context of usage counts.

Table 3 presents distributions of “poorly cited”, “fairly cited”, “remarkably cited”, and “outstandingly cited” papers in the six disciplines which we considered in our study. The statistics in the table refer to CSS scores across 16 citing years (beginning in 2000). For example, the mean percentage of poorly cited papers in natural sciences is 70.57% across 16 citing years; the lowest percentage is 66.21% and the highest is 77.49%. The range between the minimum and maximum percentages is 11.28 points. The comparison of the percentages in Table 3 with the general distribution pattern of percentages (70—21—7—2%) reveals that natural sciences, engineering and technology, medical and health sciences, and agricultural sciences are more similar to the general distribution pattern than the social sciences and the humanities. However, the largest variability of the percentages over the years can be observed for the agricultural sciences (see the ranges in Table 3).

**Table 3:** Percentages of papers across four citation impact classes published in six disciplines (in percent)

	Mean	Min	Max	Range
Natural sciences				
Poorly cited	70.57	66.21	77.49	11.28
Fairly cited	20.94	16.8	23.53	6.74
Remarkably cited	6.05	3.71	7.21	3.5
Outstandingly cited	2.44	1.69	3.38	1.69
Total	100.00	100.00	100.00	100.00
Engineering and technology				
Poorly cited	72.92	69.1	86.32	17.21
Fairly cited	19.64	9.95	22.57	12.62
Remarkably cited	5.41	2.5	6.55	4.05
Outstandingly cited	2.03	1.23	2.47	1.23
Total	100.00	100.00	100.00	100.00
Medical and health sciences				
Poorly cited	68.51	63.42	80.13	16.72
Fairly cited	22.47	12.68	27.41	14.73
Remarkably cited	6.36	4.63	7.82	3.18
Outstandingly cited	2.66	1.84	3.57	1.73
Total	100.00	100.00	100.00	100.00
Agricultural sciences				
Poorly cited	69.73	60.59	87.83	27.24
Fairly cited	22.07	9.47	32.2	22.73
Remarkably cited	6.48	1.86	7.68	5.82
Outstandingly cited	1.73	0.84	2.02	1.18
Total	100.00	100.00	100.00	100.00
Social sciences				
Poorly cited	75.54	72.24	87.93	15.69
Fairly cited	17.13	9.01	22.61	13.6
Remarkably cited	5.22	1.95	6.14	4.18
Outstandingly cited	2.11	1.1	2.56	1.46
Total	100.00	100.00	100.00	100.00
Humanities				
Poorly cited	82.22	79.34	92.62	13.28
Fairly cited	14.45	6.09	17.36	11.28
Remarkably cited	2.61	0.99	3.45	2.46
Outstandingly cited	0.72	0.3	0.99	0.69
Total	100.00	100.00	100.00	100.00

Similar field-specific differences in distributions of CSS scores are also reported by Glänzel (2011) and Albarrán and Ruiz-Castillo (2011).

### Sequence analysis

Table 4 shows the most frequent sequences of CSS scores in the dataset and their prevalence in natural sciences, engineering and technology, medical and health science, agricultural sciences, social sciences, and humanities. We made a cut at 0.5% which means that only sequences are listed in the table with a percentage of at least 0.5 in the dataset of all publications. In order to compare disciplinary differences between the same set of sequences, the selected 17 sequences from the total set are listed for all disciplines (although other sequences might meet the threshold of 0.5% in single disciplines).

**Table 4:** Most frequent sequences in the dataset (at least 0.5%) and their prevalence in six disciplines

Sequence	Natural sciences		Engineering and technology		Medical and health sciences		Agricultural sciences		Social sciences		Humanities		Total	
	Absolute number	In percent	Absolute number	In percent	Absolute number	In percent	Absolute number	In percent	Absolute number	In percent	Absolute number	In percent	Absolute number	In percent
0000000000000000	83,862	24.22	28,698	24.16	52,182	22.84	6129	18.58	15,440	29.59	2337	19.59	188,648	23.86
0100000000000000	6735	1.94	2259	1.90	4258	1.86	1359	4.12	2146	4.11	464	3.89	17,221	2.18
1000000000000000	8726	2.52	1803	1.52	3737	1.64	323	0.98	952	1.82	230	1.93	15,771	1.99
0010000000000000	4339	1.25	2052	1.73	2303	1.01	296	0.90	781	1.50	307	2.57	10,078	1.27
0001000000000000	2937	0.85	1713	1.44	1827	0.80	297	0.90	578	1.11	268	2.25	7620	0.96
0000000000010000	2806	0.81	886	0.75	2152	0.94	232	0.70	328	0.63	194	1.63	6598	0.83
0000000000001000	2600	0.75	923	0.78	1937	0.85	180	0.55	310	0.59	225	1.89	6175	0.78
0000000000100000	1989	0.57	885	0.75	2535	1.11	181	0.55	297	0.57	202	1.69	6089	0.77
0000000000000100	2442	0.71	830	0.70	1759	0.77	176	0.53	286	0.55	203	1.70	5696	0.72
0000100000000000	2176	0.63	1098	0.92	1327	0.58	239	0.72	512	0.98	272	2.28	5624	0.71
0000000000000010	2289	0.66	814	0.69	1546	0.68	164	0.50	270	0.52	205	1.72	5288	0.67
0000000000000001	2301	0.66	779	0.66	1376	0.60	147	0.45	253	0.48	215	1.80	5071	0.64
0000010000000000	2040	0.59	923	0.78	1081	0.47	250	0.76	503	0.96	178	1.49	4975	0.63
0000000100000000	2026	0.59	760	0.64	959	0.42	211	0.64	341	0.65	223	1.87	4520	0.57
0000001000000000	1373	0.40	918	0.77	1154	0.51	254	0.77	502	0.96	159	1.33	4360	0.55
0000000001000000	1774	0.51	641	0.54	728	0.32	239	0.72	339	0.65	198	1.66	3919	0.50
0000000010000000	1719	0.50	680	0.57	855	0.37	247	0.75	361	0.69	37	0.31	3899	0.49
Total	346,318	38.15	118,781	39.28	228,502	35.76	32,987	33.12	52,182	46.37	11,928	49.61	790,698	38.14

The analysis is based on four categories: poorly cited (0), fairly cited (1), remarkably cited (2), and outstandingly cited (3). However, the most frequent sequences consist of only poorly cited (0) and fairly cited (1) elements

In accordance with the prevalence of skewed citation distributions in the sciences and the dominance of non-cited and little cited papers, the list of sequences in Table 4 only contains two CSS scores: 0 = poorly cited and 1 = fairly cited. Thus, in the set of all papers (and also in most of the disciplines), sequences with 3 = remarkably cited and 4 = outstandingly cited are rare (less than 0.5%).

**Fig. 2:**
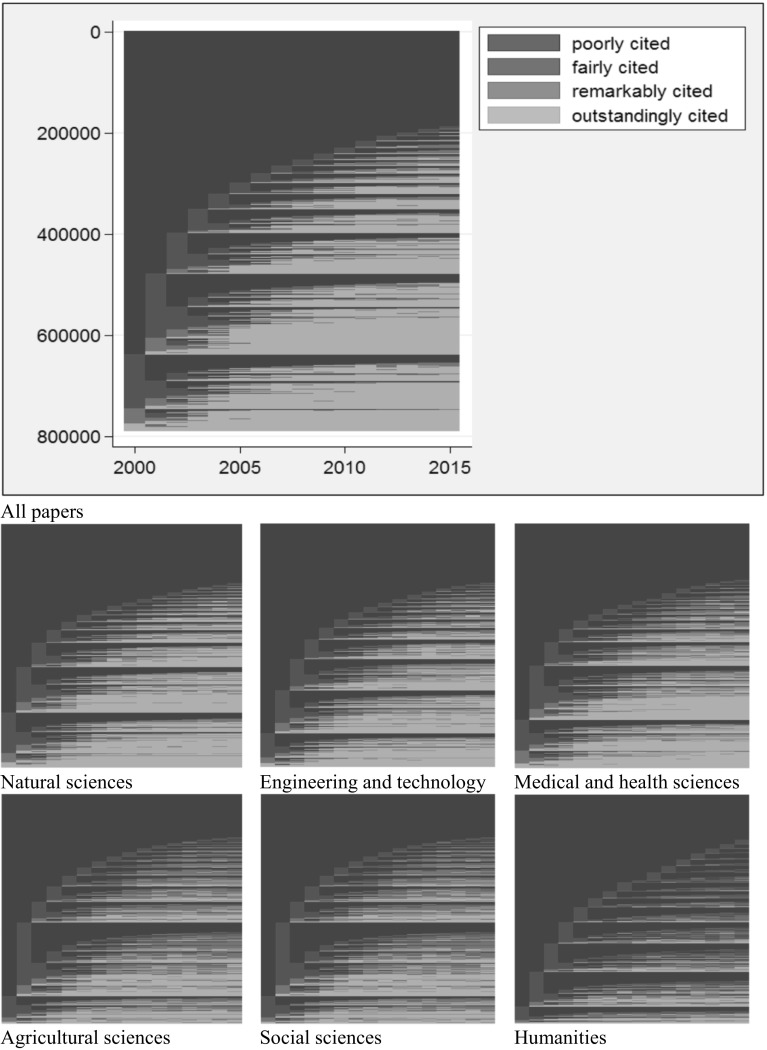
Sequence index plots for all papers (*n* = 790,698) and six disciplines

Figure 2 shows the sequences in the dataset as sequence index plots. Whereas Table 4 focusses on the most frequent sequences, all sequences are included in Fig. 2. The plots show a horizontal line for each sequence, distinguishing the CSS scores with different colors (Brzinsky-Fay et al. 2006). Similarly to Table 4, Fig. 2 demonstrates that the group of sequences with constantly poorly cited elements is the biggest group at the top of the plots. Below this biggest group, we can observe those sequences which are commonly labeled as sleeping beauties. This is a relatively small set of papers which are poorly cited initially and remarkably or outstandingly cited in later years. Another group of papers (sequences) is also clearly visible in Fig. 2. These papers are poorly cited most of the time with a short interruption of a fairly cited period (mostly 1 year). The probability of interruption in early years is higher than in later years in all disciplines. This is especially visible for the agricultural sciences and social sciences, where a large red bar is visible in the second year after publication (see the corresponding higher percentages for these disciplines in Table 4). At the bottom of all plots, the small set of constantly outstandingly papers is visible.

With regard to the differences between the disciplines, Table 4 shows that the social sciences are the discipline with the highest percentage of constantly poorly cited papers (29.59%). The lowest percentages are in the agricultural sciences (18.58%) and humanities (19.59%). Thus, here is a large difference between the social sciences and the humanities (although they are frequently treated together in bibliometrics). However, both disciplines show similar results, if we look at the horizontal “Total” line in Table 4. Both disciplines have the highest percentages, which mean that the sequences are more highly concentrated than those in other disciplines. This might be partly an effect of the lower number of sequences. However, agricultural sciences also have a relatively low number of sequences, but the concentration of sequences is significantly lower than in the social sciences and the humanities.

In order to obtain a better overview of the sequences in the dataset, two further analyses have been done. The analyses condense the sequences still further. The first condensation which is shown in Table 5 treats CSS scores identically if they consist of the same elements. That means the sequence $\left\{ {2112} \right\}$ is treated the same as $\left\{ {1222} \right\}$ because both sequences consist of the CSS scores 2 and 1 only. The results in Table 5 refer to the complete dataset and are not restricted to the most frequent sequences unlike the results in Table 4. The results in Table 5 confirm the results in Table 4 and Fig. 2. About a quarter of the sequences consist of constantly poorly cited papers $\left\{ 0 \right\}$. However, the largest group of sequences $\left\{ {01} \right\}$ is that which includes poorly cited and fairly cited periods (46.85%). This group of papers is especially dominant in the humanities with 64.35%. There is a third large group of sequences (19.43%) in Table 5 $\left\{ {012} \right\}$ which includes poorly cited, fairly cited, and remarkably cited periods. This group contains about 20% of the papers in all disciplines except one: in the humanities, only 11.82% of the papers have these three elements.

**Table 5:** Sequences consisting of the same elements by disciplines

Sequence-elements	Natural sciences		Engineering and technology		Medical and health sciences		Agricultural sciences		Social sciences		Humanities		Total	
	Absolute number	In percent	Absolute number	In percent	Absolute number	In percent	Absolute number	In percent	Absolute number	In percent	Absolute number	In percent	Absolute number	In percent
01	160,183	46.25	56,126	47.25	107,098	46.87	15,823	47.97	23,539	45.11	7676	64.35	370,445	46.85
0	83,862	24.22	28,698	24.16	52,182	22.84	6129	18.58	15,440	29.59	2337	19.59	188,648	23.86
012	66,729	19.27	23,114	19.46	45,729	20.01	7915	23.99	8743	16.75	1410	11.82	153,640	19.43
0123	20,953	6.05	7365	6.20	13,670	5.98	2300	6.97	2840	5.44	355	2.98	47,483	6.01
123	6342	1.83	1112	0.94	4535	1.98	274	0.83	514	0.99	21	0.18	12,798	1.62
013	1825	0.53	663	0.56	724	0.32	213	0.65	297	0.57	33	0.28	3755	0.47
12	1528	0.44	186	0.16	944	0.41	33	0.10	71	0.14	3	0.03	2765	0.35
023	1392	0.40	490	0.41	919	0.40	100	0.30	214	0.41	9	0.08	3124	0.40
23	1320	0.38	199	0.17	1145	0.50	22	0.07	100	0.19	7	0.06	2793	0.35
02	1131	0.33	580	0.49	791	0.35	149	0.45	243	0.47	67	0.56	2961	0.37
3	417	0.12	65	0.05	383	0.17	6	0.02	37	0.07	1	0.01	909	0.11
03	340	0.10	129	0.11	211	0.09	18	0.05	102	0.20	9	0.08	809	0.10
13	266	0.08	54	0.05	162	0.07	5	0.02	41	0.08	0	0.00	528	0.07
1	27	0.01	0	0.00	9	0.00	0	0.00	1	0.00	0	0.00	37	0.00
2	3	0.00	0	0.00	0	0.00	0	0.00	0	0.00	0	0.00	3	0.00
Total	346,318	100.00	118,781	100.00	228,502	100.00	32,987	100.00	52,182	100.00	11,928	100.00	790,698	100.00

The analysis is based on four categories: poorly cited (0), fairly cited (1), remarkably cited (2), and outstandingly cited (3)

The results in Table 5 allow a closer look at the sequences which include outstandingly cited periods (3). The largest group of papers with such a period is $\left\{ {0123} \right\}$ (6.01%), which is followed by $\left\{ {123} \right\}$ (1.62%) in the table. Only 0.11% of the papers (*n* = 909) are constantly on the outstandingly cited level over a period of 16 years. Most of these papers have been published in the natural sciences (*n* = 417) and medical and health sciences (*n* = 383). There is only one such paper in the humanities and 6 such papers in agricultural sciences. Constant performers on the level of fairly cited (1) or remarkably cited (2) are very rare in the dataset. In total, only 37 papers are constantly fairly cited and 3 papers constantly remarkably cited.

The second condensation which is shown in Table 6 treats identically all sequences that have the same order of CSS scores. That means the sequence $\left\{ {2112} \right\}$ is treated the same as $\left\{ {211112} \right\}$ because the CSS scores appear in the same order in both sequences (first 2, then 1, and then 2 again). The sequences which are shown in Table 6 are restricted to those with at least 0.5% of the papers in the dataset—similar to Table 4. Again, the results in Table 6 reveal that about a quarter of the papers are constantly poorly cited (with a significantly higher percentage in the social sciences). 13.9% of the papers have a sequence with initially increasing citation impact (from 0 to 1) and then decreasing (from 1 to 0). For 8.66 and 5.51% of the papers the $\left\{ {010} \right\}$ sequence order is followed by a $\left\{ {10} \right\}$ and $\left\{ {1010} \right\}$ sequence.

**Table 6:** Most frequent sequences with elements in the same order in the dataset (at least 0.5%) and their prevalence in six disciplines

Sequence-order	Natural sciences		Engineering and technology		Medical and health sciences		Agricultural sciences		Social sciences		Humanities		Total	
	Absolute number	In percent	Absolute number	In percent	Absolute number	In percent	Absolute number	In percent	Absolute number	In percent	Absolute number	In percent	Absolute number	In percent
0	83,862	24.22	28,698	24.16	52,182	22.84	6129	18.58	15,440	29.59	2337	19.59	188,648	23.86
010	44,975	12.99	18,638	15.69	29,350	12.84	4929	14.94	8546	16.38	3451	28.93	109,889	13.90
01010	27,913	8.06	11,309	9.52	19,917	8.72	3322	10.07	4550	8.72	1497	12.55	68,508	8.66
0101010	17,362	5.01	6781	5.71	13,720	6.00	2171	6.58	2781	5.33	728	6.10	43,543	5.51
10	11,565	3.34	2282	1.92	4956	2.17	486	1.47	1182	2.27	256	2.15	20,727	2.62
010101010	8189	2.36	2994	2.52	6784	2.97	1191	3.61	1281	2.45	268	2.25	20,707	2.62
010101	7243	2.09	2630	2.21	5392	2.36	663	2.01	970	1.86	296	2.48	17,194	2.17
01010101	5970	1.72	2087	1.76	4745	2.08	598	1.81	844	1.62	227	1.90	14,471	1.83
1010	7242	2.09	1487	1.25	3546	1.55	380	1.15	555	1.06	116	0.97	13,326	1.69
0101	5722	1.65	1991	1.68	3922	1.72	402	1.22	667	1.28	257	2.15	12,961	1.64
101010	5327	1.54	1092	0.92	2836	1.24	260	0.79	378	0.72	59	0.49	9952	1.26
01	3189	0.92	1094	0.92	1845	0.81	185	0.56	322	0.62	257	2.15	6892	0.87
10101010	3413	0.99	727	0.61	1917	0.84	180	0.55	219	0.42	40	0.34	6496	0.82
0101010101	2596	0.75	877	0.74	2191	0.96	324	0.98	410	0.79	81	0.68	6479	0.82
01010101010	1995	0.58	730	0.61	1880	0.82	332	1.01	292	0.56	63	0.53	5292	0.67
Total	346,318	68.31	118,781	70.23	228,502	67.91	32,987	65.33	52,182	73.66	11,928	83.27	790,698	68.94

The analysis is based on four categories: poorly cited (0), fairly cited (1), remarkably cited (2), and outstandingly cited (3). However, the most frequent sequences consist of only poorly cited (0) and fairly cited (1) elements

In Table 6, remarkably cited or outstandingly cited periods do not play any role. Their occurrences are too low in general.

## Discussion

In recent years, a development has become apparent in bibliometrics for citation impact no longer to be reduced to the times cited information, but analyzed more specifically. For example, the citation context is considered in the bibliometric analyses to have more specific information on the impact of publications and how cited publications are perceived (Small et al. 2017). Carroll (2016) takes into account “the frequency with which the paper is cited within citing publications … adding depth and value to the citation metric” (p. 1329). The results of Hu et al. (2015) show that successive citations in papers are more intentional and reasonable than first-time citations—if papers are cited multiple times in a paper. The “Literature overview” section in this paper presents some further studies which take a closer look at citations by investigating the citation history of papers.

In this study, we used a method for the analysis of citation distribution which has never been used before in bibliometrics (to the best of our knowledge). Based on annually normalized citation scores, we generated annual sequences of CSS scores (e.g. $\left\{ {01233233221} \right\}$) which we analyzed using the strategy proposed by Brzinsky-Fay et al. (2006). This strategy allows the identification of very frequent and less frequent sequences over the complete publication set and disciplinary sets. In agreement with previous studies, our results demonstrate that sequences with poorly cited (0) and fairly cited (1) elements dominate the publication set; sequences with remarkably cited (3) and outstandingly cited (4) periods are rare. The highest percentages of constantly poorly cited papers can be found in the social sciences; the lowest percentages are in the agricultural sciences and humanities. The largest group of papers with remarkably cited (3) and/or outstandingly cited (4) periods shows an increasing impact over the citing years with the following orders of sequences: $\left\{ {0123} \right\}$ (6.01%), which is followed by $\left\{ {123} \right\}$ (1.62%). Only 0.11% of the papers (*n* = 909) are constantly on the outstandingly cited level. These might be the few papers which significantly drive scientific progress (Rodríguez-Navarro 2016).

This study was a first attempt to use sequence analyses with bibliometric data. We think that this statistical approach can lead to interesting insights in citation histories. The application of this approach can be further extended beyond the analyses in our study. For example, a focus of future research could be on the comparison of sequences and the measurement of differences between two sequences. According to Brzinsky-Fay et al. (2006), the so-called Levenshtein distance has been used for comparisons in various fields, such as plagiarism detection and the analysis of DNA sequences. The Levenshtein distance quantifies the distance between two sequences. Another topic for future research could be possible explanations of differences between sequences. Distance measures between two sequences could be included as dependent variables in regression models, which are then explained by various characteristics of the publications (e.g., their subject category, country of origin, or reputations of authors).
